# Need analysis for a new high acuity medical psychiatry unit: which patients are considered for admission?

**DOI:** 10.1186/s12913-019-3967-7

**Published:** 2019-02-28

**Authors:** P. J. Caarls, M. A. van Schijndel, M. Kromkamp, A. I. Wierdsma, R. J. Osse, G. van der Hoeven, W. J. G. Hoogendijk, J. J. van Busschbach

**Affiliations:** 1 Erasmus MC, University Medical Center Rotterdam, Department of Psychiatry, s-Gravendijkwal 230, 3015 CE Rotterdam, The Netherlands; 2 Rijnstate Hospital, Department of Psychiatry, Wagnerlaan 55, 6815 AD Arnhem, The Netherlands; 3 University Medical Center Utrecht, Department of Psychiatry, Heidelberglaan 100, 3584 CX Utrecht, The Netherlands

**Keywords:** Medical psychiatry unit, Comorbidity, Hospital organization, Collaborative care, Patient admission

## Abstract

**Background:**

The study aims were: to estimate the proportion of patients with an indication for admission to a new high acuity Medical Psychiatric Unit (MPU), to explore the reasons for MPU-admission according to different health disciplines, and to check for differences in patient characteristics. The results of this study are to be utilized in the proposed establishment of a high-acuity MPU in a University Medical Center. Such a unit currently does not exist at Erasmus MC.

**Methods:**

Hospital in-patients were included if they received psychiatric consultation from the Psychiatric Consultative Service (PCS). As part of the study protocol, psychiatrists, other medical specialists, and nurses determined the need for admission to the proposed MPU. Patient groups were compared with respect to diagnoses, socio-demographic characteristics and patient routing.

**Results:**

One hundred and fifty-one patients were included, 43% had an indication for MPU-admission, for the other patients PCS involvement was sufficient. There was agreement on suicide attempts as a reason for MPU-admission. For psychiatrists, the need for further diagnostic evaluation was a common reason for MPU admission, while other medical specialists more often emphasized the need for safety measures. Patients with an unplanned hospital admission had a higher chance of MPU eligibility (OR = 2.72, 95% CI 1.10–6.70). The main psychiatric diagnoses of MPU-eligible patients were organic disorders (including delirium), mood disorders, and disorders related to substance abuse. The most common diagnoses found were similar to those in previous research on MPU populations.

**Conclusion:**

Different medical disciplines have different views on the advantages of MPUs, while all see the need for such facilities. The proposed MPU should be able to accommodate patients directly from the Emergency Unit, and the MPU should provide specialized diagnostic care in an extra safe environment.

## Background

High acuity Medical Psychiatry Units (MPUs) add value through their ability to institute aggressive, combined treatment for patients with complicated physical health and mental health/substance use disorders in the general-medical hospital setting. These units, also known as Complexity Intervention Units, have moderate-to-high physical health and mental health/substance abuse acuity capabilities [1]. MPUs close the gap between psychiatric wards, that are usually not equipped to deal with more than minimal medical or surgical problems, and medical wards that often have limited tolerance for patients with a psychiatric disorder or behavioral problems [2–4]. These units are complementary to (pro-active) psychiatric consultation services when biopsychosocial complexity is severe [1, 2, 5].

Erasmus MC, a large University Medical Center in Rotterdam, the Netherlands, aims to inaugurate an MPU in 2019. The reasons for establishing such a unit fall into several categories: 1) managing the increasing complexity and behavior that is disruptive to somatic treatment in an aging and more often multimorbid population [6–9], 2) improving the quality of care [9], and 3) the promise of cost-effectiveness by reducing length of stay and readmissions [8, 10].A needs assessment is generally advised before establishing such a unit [11, 12].

MPUs appear in many forms. Some are able to treat the most severe patients, while others can only deal with light forms of somatic and/or psychiatric illness. Kathol devised a two-axes classification system to describe the different types of MPU, with one axis describing the psychiatric illness and one the somatic illness [5]. Which type of MPU is required depends on the needs of the patient in a particular hospital [5, 13]. Other locally influential factors are available staff, funding, and collaboration with other hospital wards [14–16]. However, there is limited consensus on MPU-admission criteria and the target population [10, 17]. The aim of this study was thus to investigate the perceived need - according to medical professionals - for an MPU in the context of a large university hospital. We investigated how potential MPU-patients differed from the group that was indicated for psychiatric consultation. Such a systematic assessment has not been described before.

## Methods

Erasmus MC, University Medical Center is a 1000-bed tertiary university referral center in Rotterdam, the Netherlands. It has many different wards, including a psychiatric ward with 57 beds. The Psychiatric Consultation Service (PCS) provides psychiatric diagnosis and treatment for medical inpatients. The PCS is staffed by a 0.8 full-time equivalent (FTE) research nurse, two 0.9 FTE residents and a 0.9 FTE psychiatrist, and has 24/7 coverage. The main senior Consultation-Liaison (C-L) psychiatrist (author RO) had been working with this study population for 15 years and the research nurse worked at the PCS for 10 years, with extensive previous experience as a psychiatric nurse. Maximum frequency of patient contact is once a day. If necessary, the consultative nurse can provide extra support and coaching of the medical nursing team.

From 2019 onwards, a new 7-bed MPU will cater for the most complex in-patients with concurrent medical and psychiatric needs. It will occupy part of one of the Internal Medicine floors. Medical staff will include an internist and a psychiatrist using a co-attending model: admission will be a joint decision made by the attending internist and the psychiatrist, every patient being admitted under the care of the internist [18–21]. The psychiatrist will be given full clinical authority for psychiatric diagnosis, psychiatric treatment and the ward milieu. Two designated internists and two designated psychiatrists will alternate as attending supervisors. House staff will consist of a psychiatric resident and internal medicine resident. The nurses will have a medical background with cross-training in psychiatric nursing.

### Patients

All patients treated by the PCS between February 1 and June 30 in 2014 were asked consent for participation in this study after discharge from the hospital. MPU-eligibility status was retrospectively assessed by employing a structured survey amongst attending physicians, consultation-liaison psychiatrists and senior nurses. Subsequently, groups were compared with respect to their medical and patient routing according to their MPU-eligibility status: MPU-eligible or PCS-only. Patients could only be included if at least a psychiatrist or another medical specialist had given their opinion on the MPU-eligibility of the respective patient. Patients who refused to participate were excluded.

### Protocol

The attending medical specialist, C-L psychiatrist, and a senior ward nurse were asked their opinions on MPU-eligibility in retrospect. Both medical specialists and nurses were surveyed as these parties are most relevant to the admission-decision process. In the absence of the main senior C-L psychiatrist, another experienced psychiatrist colleague was asked for his/her opinion.

### Measures

All opinions on MPU eligibility were registered on a standard form that gave a general definition of the proposed MPU: “The MPU will have the ability to care for patient physical needs as on a normal hospital ward and will have the ability to care for patient psychiatric needs as on a standard psychiatric hospital ward.” When one of the doctors saw an indication for MPU-eligibility, the patient was included in the MPU-eligible group. In general, the doctors and nurses decided on MPU-eligibility by considering there to be a need for both psychiatric admission and general hospital admission.

Socio-demographic data (age, marital status and country of birth), medical data, and patient routing were obtained from the electronic medical record and hospital information system retrospectively. Medical data contained the primary reason for admission, somatic main diagnosis, and psychiatric main diagnosis. ICD-10 chapter codes were used to describe the diagnoses and reasons for admission. Patient routing data consisted of: the type of referral, number of days until psychiatric consultation, the specialism requesting consultation, number of days in normal, medium, high and intensive care, length of stay, discharge destination, and readmissions until 22nd July 2014.

### Statistical analyses

Data were reported for two groups according to MPU eligibility status: MPU-indication versus PCS-only. Univariate associations between admission decision and demographic and clinical covariates were analyzed using standard statistical tests: chi-square test for categorical variables and t-test or Mann-Whitney U test for interval- or ratio-level data. Following Hosmer and Lemeshow’s approach [22], multiple logistic regression analyses were performed using stepwise forward and backward procedures with 0.25 and 0.05 alpha levels of entry and removal respectively. Interaction effects and collinearities were checked for all significant main factors. Model selection was based on likelihood ratio test statistics. This stepwise model selection procedure is appropriate for the explorative nature of this study. The fit of the final model was assessed using Nagelkerke R2, the ROC curve, and the Hosmer-Lemeshow goodness-of-fit test. SPSS for Windows (version 21) was used to perform all statistical procedures.

## Results

**Fig. 1:**
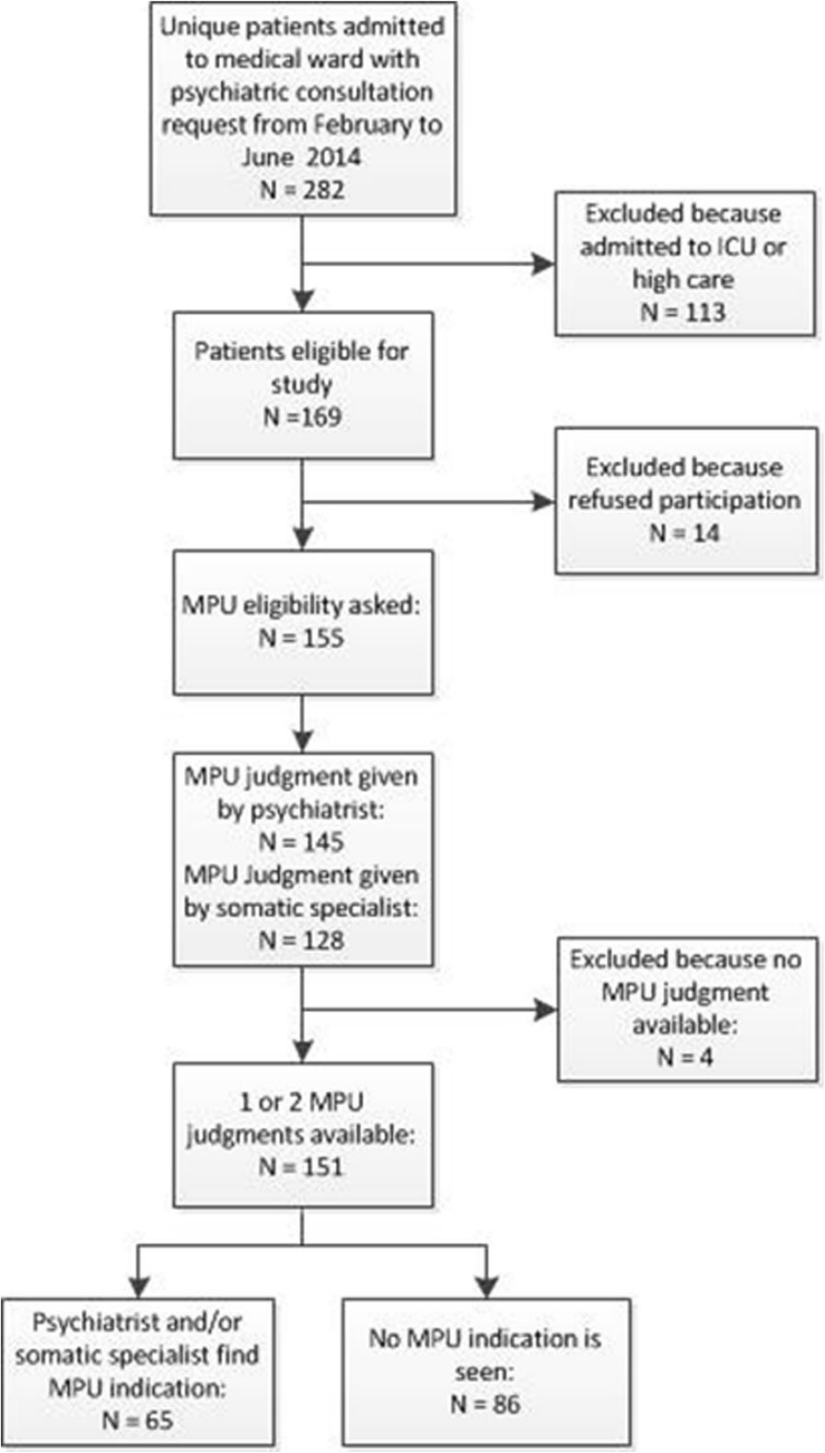
flowchart of inclusion and exclusion

We enrolled 151 patients in the study; 86 (57%) were allocated to the PCS-only arm. Sixty-five patients (43%) were allocated to the potential MPU, for 37 of whom the C-L psychiatrist and medical/surgical specialist agreed on MPU-eligibility. The total agreement of eligibility (both MPU-eligibility and PCS-only eligibility) between medical/surgical attending personnel and the C-L psychiatrist occurred in 77% (*n* = 116) of the cases. Figure 1 shows the flow of inclusion and exclusion of patients.

Sixty different specialists from 12 different specialisms were surveyed: 16 surgeons, 14 internists, 5 Ear, Nose, Throat specialists, 4 neurologists, 3 neurosurgeons, 3 gynecologists, 3 hematologists, 3 urologists, 2 pulmonologists, 2 geriatrists, 1 cardiologist, and 4 psychiatrists. The medical/surgical specialists and ward nurses had varying levels of experience. For the psychiatrists the time between a consultation request and a decision on MPU-eligibility was a median of 8 days (range 0 to 79 days), for the somatic specialists the median was 66.5 days (range 0 to 178 days).

In general, a patient was perceived as an eligible MPU-patient if this patient had both: a) an indication for a psychiatric hospital admission based on moderate or severe psychiatric symptoms and the need for treatment of these symptoms, or behavioral problems in a psychiatric hospital (for instance due to suicidal behavior, or lack of symptom control making ambulatory psychiatric treatment difficult), and b) an indication for general hospital admission based on moderate or severe somatic medical symptoms.

In the cases of agreement between medical/surgical attending personnel and the C-L psychiatrist, the main reasons for MPU-eligibility were suicide attempts and concurrent general medical and psychiatric illnesses (for example patients with HIV or Cushing disorder). The arguments of the C-L psychiatrist for MPU-eligibility focused more on the need for a diagnostic evaluation, while the medical/surgical specialist’s motivation was more often based on a lack of expertise of psychiatric problems, or safety issues such as wandering behavior and suicidal behavior, and the consequent need for a closed ward. Agreement on MPU non-eligibility was seen mostly in patients with too severe medical problems, and the need for special facilities (i.e. isolation room, specialist oncology ward, or after complex cardiologic or neurologic operations). Furthermore, end-of-life care, non-complex delirium, no psychiatric disorder, and consultative services on a regular medical/surgical ward, were agreed reasons for no need for MPU admission. In situations with disagreement on MPU eligibility, it varied whether the psychiatrist or medical specialist would prefer MPU admission. For 13 patients (9%) only a medical specialist preferred MPU admission, and for another 13 patients (9%) only the psychiatrist preferred MPU admission. In 6 cases the medical specialist stated that the patient could maintain himself on their ward, while the psychiatrist saw the benefit of MPU admission.

A remarkable disagreement was seen for 4 patients who were admitted to the hospital because of suicide attempts. A particular internal medicine specialist stated that during the short time these patients were admitted, for none of these patients MPU admission would be necessary. The psychiatrist, and most other medical specialists, recommended MPU admission for around 90% of patients with suicide attempts.

For the other disagreements no distinct patterns appeared. For 3 cases the C-L psychiatrist or the other medical specialist involved suggested that geriatric admission would be more appropriate. For 49 patients (79%) with an indication for MPU admission according to either the psychiatrist or the other medical specialist, the nurse agreed with the physician on the MPU indication. The motivation of the nurse matched the motivation of the physician(s) for: MPU admission for patients with suicide attempts and suicidal behavior, concurrent general medical and psychiatric illness, and the lack of wards’ expertise to handle severe psychiatric problems. Nurses more often stated psychiatric history and (prominent) behavioral, cognitive or psychiatric symptoms as reasons for MPU eligibility. Nurses also more often stated ‘acts like any other patient’, no psychiatric symptoms, or no difficulties in handling behavioral problems, as reasons for non-necessary MPU admission. When physicians saw no indication for MPU admission, for 25 patients (36%) nurses did see an MPU indication. Reasons for admission according to nurses only were delirium, psychological aspects, treatment refractoriness, substance abuse and paranoia, suicidal behavior, prophylactic treatment, psychiatric comorbidity and treatment adherence problems. Table 1 shows that patients with an MPU indication were on average 8 years younger. There were no significant socio-demographic differences between the two groups.

**Table 1:** Socio-demographic characteristics

	Indication for MPU (*N* = 65)		PCS-only (*N* = 86)		*P* value
	N	%	N	%	
Female	27	42%	33	38%	.694
Age (mean ± sd)	47.9 ± 16.4		56.2 ± 16.2		.002
Marital status:					.105
- married	20	31%	33	38%	
- never married	24	37%	20	26%	
- widowed	0	0%	5	6%	
- divorced	12	19%	10	12%	
- unknown	9	14%	19	19%	
Place of birth					.536
- Netherlands	41	63%	58	68%	
- foreign	16	25%	15	17%	
- unknown	8	12%	13	15%	

### Patient routing

Table 2 shows the routing of patients through the hospital. Patients with an indication for MPU admission were mostly admitted via the emergency room (*N* = 46, 71%). Of the patients with a need for MPU admission, about one third was admitted at an internal medicine ward. Neurology was the second referring specialism for patients with a need for MPU admission, followed by traumatology and general surgery. The patients with an indication for MPU admission were visited for psychiatric consultation sooner (median 1 day versus 5 days, *P* < .001). Patients without an indication for MPU admission had a longer length of stay (LOS) at the hospital (median 15 days versus 6 days, *P* = .003).

**Table 2:** Patient routing

	MPU-eligible (*N* = 65)		PCS-only (*N* = 86)		*P*-value
Hospital entry					*P* = .015
- emergency room	46	71%	38	44%	
- from home	3	5%	7	8%	
- outpatient clinic	11	17%	26	30%	
- referred from other hospital	5	8%	14	16%	
- other	0	0%	1	1%	
Days until psychiatric consultation, median (min-max)	1	0–84	5	0–51	*P* = .000
Specialism requesting consult					*P* = .002
- internal medicine	22	34%	11	13%	
- general surgery	6	9%	14	16%	
- gastroenterology	5	8%	10	12%	
- traumatology	7	11%	8	9%	
- neurology	9	14%	2	2%	
- neurosurgery	2	3%	8	9%	
- ear, nose, throat	0	0%	10	12%	
- other	14	27%	23	27%	
Number of wards per admission, median (min-max)	1	1–8	1	1–10	NA
Admission on ICU, %	9	14%	23	27%	*P* = .046
Days at ICU, median (min-max)	0	0–13	0	0–54	NA
Admission on high care, %	1	1%	4	5%	NA
Days at high care, median (min-max)	2	–	0	0–11	*P* = .532
Admitted at medium care, %	0	0%	3	4%	NA
Days at medium care, median (min-max)	–	–	0	0–30	NA
Total length of stay, median days (min-max)	6	1–104	15	0–116	NA
Disposition location					NA
- home	44	68%	46	54%	
- nursing home	2	3%	14	16%	
- revalidation	1	2%	6	7%	
- other hospital	1	2%	2	2%	
- psychiatric hospital	8	12%	0	0%	
- died	4	6%	11	13%	
- other	5	8%	6	7%	
Readmission within 1 month	5	9%	20	27%	NA

*ICU* intensive care unit. For readmission within 1 month 129 patients could be included in analysis. If the number of patients was too low no statistical test was performed, in the table this is shown as (non-applicable, NA)

MPU eligible patients were more often discharged to a psychiatric hospital.

### Medical data

Table 3 shows that almost half of the patients with an indication for MPU admission stayed in Erasmus MC because of injury or poisoning. The same trend was seen in the final diagnosis at discharge.

**Table 3:** Medical data

Admission reason (%):	Indication for MPU		PCS-only	
	(*N* = 64)		(*N* = 86)	
External causes of morbidity and mortality, XX	31	48%	19	22%
- Accident	4	6%	8	9%
- Intentional self-harm	21	32%	1	1%
Injury, poisoning and certain other consequences of external causes, XIX	30	46%	16	19%
- Injury of multiple body regions	4	6%	6	7%
- Poisoning by drugs, medicaments and biological substances	14	22	1	1%
Neoplasms, II	9	14%	20	23%
Symptoms, signs and abnormal clinical and laboratory findings, XVIII	12	19%	17	20%
Diseases of the digestive system, XI	6	9%	12	14%
Diseases of the circulatory system, IX	4	6%	11	13%
Certain infectious and parasitic diseases, I	4	6%	7	8%
Diseases of the nervous system, VI	3	5%	6	7%
Diseases of the genitourinary system, XIV	5	8%	1	1%
Mental and behavioral disorders, V	3	5%	1	1%
Diseases of the skin and subcutaneous tissue, XII	1	2%	3	4%
Pregnancy, childbirth and the puerperium, XIV	1	2%	3	4%
Diseases of the respiratory system, X	1	2%	2	2%
Endocrine, nutritional and metabolic diseases, IV	1	2%	1	1%
Diseases of the musculoskeletal system and connective tissue, XIII	2	3%	0	0%
Diseases of the blood and blood-forming organs and certain disorders involving the immune mechanism, III	1	2%	0	0%
Diseases of the eye and adnexa, VII	0	0%	1	1%
Primary somatic conclusion (%)				
External causes of morbidity and mortality, XX	41	59%	26	30%
- Accident	4	6%	9	11%
- Intentional self-harm	24	37%	1	1%
- Medical	6	9%	17	18
Injury, poisoning and certain other consequences of external causes, XIX	35	54%	18	21%
Neoplasms, II	10	15%	21	24%
Symptoms, signs and abnormal clinical and laboratory findings, XVIII	12	19%	16	19%
Diseases of the digestive system, XI	7	11%	19	22%
Certain infectious and parasitic diseases, I	5	8%	15	17%
Diseases of the circulatory system, IX	4	6%	16	19%
Diseases of the nervous system,	8	12%	9	11%
Diseases of the respiratory system, X	1	2%	15	17%
Diseases of the skin and subcutaneous tissue, XII	1	2%	10	12%
Diseases of the genitourinary system, XIV	5	8%	6	7%
Endocrine, nutritional and metabolic diseases, IV	2	3%	4	5%
Pregnancy, childbirth and the puerperium, XV	2	3%	3	4%
Diseases of the ear and mastoid process, VIII	1	2%	1	1%
Diseases of the musculoskeletal system and connective tissue, XIII	1	2%	1	1%
Factors influencing health status and contact with health services, XXI	1	2%	1	1%
Diseases of the blood and blood-forming organs and certain disorders involving the immune mechanism, III	1	2%	0	0%
Diseases of the eye and adnexa, VII	0	0%	1	1%
Psychiatric conclusion, V (%)				
Any psychiatric conclusion	59	91%	69	80%
Organic, including symptomatic, mental disorders	16	25%	44	51%
Mood [affective] disorders	9	14%	8	9%
Mental and behavioral disorders due to psychoactive substance use	8	12%	3	4%
Disorders of adult personality and behavior	8	12%	3	4%
Neurotic, stress-related and somatoform disorders	4	6%	5	6%
Schizophrenia, schizotypal and delusional disorders	7	11%	1	1%
Behavioral syndromes associated with physiological disturbances and physical factors	1	2%	1	1%
Disorders of psychological development	0	0%	1	1%
Behavioral and emotional disorders with onset usually occurring in childhood and adolescence	0	0%	1	1%
Unspecified mental disorder	6	9%	2	2%

Diagnoses ordered from most frequent to least frequent. Roman numbers behind diagnoses correspond with ICD chapter code. ICD-10 subcategories were explored for the two categories wherein most patients were included. If someone had a diagnosis in chapter “External causes of morbidity and mortality” a diagnosis in another chapter was also required, usually in chapter “Injury, poisoning and certain other consequences of external cause”. This resulted in many patients having at least two diagnoses. Psychiatric conclusions described with ICD-10 blocks of chapter V mental and behavioral disorders (F00-F99)

A psychiatric diagnosis was found in a large majority of both groups. For both groups the most frequent psychiatric diagnosis was organic mental disorder, including delirium. Mood disorder was the second most frequent diagnosis, both in the MPU and the PCS-only groups. Disorders due to substance abuse were more often seen in patients with an MPU indication.

Logistic regression analysis explored the relationship between the MPU eligibility verdict and socio-demographic variables, hospital routing data and medical data. The results indicated that psychiatric conclusion and hospital entry were independent explanatory factors (Nagelkerke R2 = 0.266; Hosmer and Lemeshow test = 3.370, degrees of freedom = 6, *p* = 0.761; Area under the Curve = 0.751, 95% Confidence Interval 0.671–0.831). Psychiatric conclusion was grouped into four categories (no psychiatric diagnosis, organic mental disorders, mood disorders and other disorders) because some diagnostic groups were too small to be taken into account separately. The odds for MPU eligibility were higher for patients with a mood disorder (OR = 4.62, 95%CI 1.12–19.08) and other psychiatric disorder (substance use, personality disorder, stress-related and somatoform disorders, schizophrenia and unspecified mental disorder (OR 5.85, 95% CI 1.86–18.42)), compared to no psychiatric conclusion. Furthermore, odds for MPU eligibility were higher for patients who would be transferred from the Emergency Room (OR = 2.72, 95% CI 1.10–6.70), compared to hospital admission after an outpatient visit.

## Discussion

Although there are some studies comparing patients admitted to a MPU with non-MPU admitted patients [7, 23], this is the first study describing the perceived need - according to professionals - for a high acuity Medical Psychiatry Unit (MPU) within a PCS patient group in a hospital without an actual MPU. Professional medical personnel pointed to a need for MPU admission in 43% of the PCS patients admitted to a normal care or medium care unit. Frequent deliberations between physicians and with nurses will occur to decide on MPU eligibility. Diagnostic opportunities were a common reason for MPU admission according to the psychiatrist, while other medical specialists more often stressed safety concerns or lack of psychiatric treatment expertise as needs for MPU admission. Hence the proposed MPU will be secluded to provide a safe environment and staff will be trained to observe behavior and provide psychiatric treatment. The MPU should be prepared to care for patients with severe psychiatric adverse effects of medications for physical conditions (e.g. dexamethasone), and for diagnostic work-up for physical conditions with psychiatric symptoms (e.g. Cushing syndrome). Furthermore, patients with an MPU indication are likely to have an unplanned admission and transfer from the Emergency Room. The MPU should thus be prepared for acute admissions. Currently, more than half of the patients with an indication for admission to the proposed MPU are admitted to an internal medicine, neurology, or traumatology ward. Together with general surgery and gastroenterology wards, these wards will refer 75% of patients with an MPU indication in the future. Support from these departments and their specialists will be crucial to establish a well-functioning MPU. Since the Erasmus MC is a tertiary referral center the MPU should be able to deliver the most complex physical and psychiatric care in order to provide added value and be acceptable for medical specialists. When both specialists agreed that there was no MPU-admission indication, they commonly stated that medical acuity was too high or specialist nursing care or equipment was needed. A Psychiatric Consultative Service should stay easily accessible for these patients to provide psychiatric assessment and treatment. Kishi et al. described the patient population of their type IV integrated medicine and psychiatry treatment program. The psychiatric conditions of their patients are similar to those of our MPU-eligible patient group, most common being substance-related disorders, delirium, mood disorders and dementia [7]. Molnar et al. explored differences between patients using a Liaison-Consultative Service (LCS) and patients on an MPU [23]. In our study organic mental disorder, including delirium, was a common diagnosis, while in Molnar’s study delirium occurred in only small number of patients. In our study substance related disorders were frequently found in the need for MPU patient group, hence the proposed MPU might need collaboration and agreements with addiction treatment centers [1].

Our results are probably influenced by the patient population in the area of the Erasmus MC, Rotterdam. Rotterdam is a large European city, with a population of 630,000 inhabitants with a multicultural background, which may contribute to the high number of patients with substance abuse disorder. The Erasmus MC also provides centralized services for trauma-patients and tertiary medical problems for the surrounding region and rural areas with approximately 2.5 million inhabitants; this might, for example, have influenced the number of patients with delirium.

The finding that patients with an indication for MPU admission received psychiatric consultation sooner is probably explained by a greater number of management problems (prominent behavioral, cognitive and psychiatric symptoms) and safety risks in this group, leading to a greater sense of urgency for psychiatric consultation. A counter-intuitive finding, when compared to previous studies, is that patients with an indication for MPU admission had a shorter LOS than patients without such an indication (median 6 days versus 15 days). In our study, medical problems that were too severe and the consequent need for special facilities was a common exclusion reason for MPU eligibility. The longer LOS of the PCS-only patients is therefore probably explained by greater medical morbidity, supported by more intensive and high care stays (27 vs 14%) and higher mortality 13 vs 6%) in this group.

Improving cost-effectiveness will be a challenge since the current length of stay without an MPU is already quite short, probably related to early PCS involvement [24, 25]. In a broader perspective, the proposed MPU may reduce the need for sequential admission in a psychiatric hospital [6, 7]. Kishi et al. found that their MPU was able to provide more improvement in psychiatric symptoms within a shorter length of stay than would have occurred with traditional sequential care [7]. Hence, direct MPU admission is recommended, to avoid provider delay [7, 26].

### Limitations and suggestions for further research

This pilot study has several limitations. First, MPU eligibility status was assessed in retrospect. Patients were considered MPU-eligible if either the medical specialist or the psychiatrist judged this to be the case. In future studies, MPU eligibility should be determined more consistently with the actual clinical process, by determining MPU eligibility repeatedly and in a dialogue between the C-L psychiatrist and the medical/surgical specialist, and to avoid recall bias. Second, although a definition of the proposed MPU was provided, it is possible that different specialists had different views with respect to the possibilities of the MPU.

An advantage of our study is that the results could not be influenced by factors such as the scarcity of MPU beds or the political pressure to admit ‘difficult’ patients. These factors however will be relevant during daily clinical practice. Another concern is the generalizability of results due to missing patients in various categories. First, there is a group of patients not visited by the PCS who might nevertheless benefit from MPU admission. This group includes patients with a known psychiatric disorder who could benefit from integrated care on an MPU, even though their behavior did not trigger a request for psychiatric consultation. This group of patients could be included if hospital admissions were screened proactively for the need for psychiatric consultation [1, 27, 28]. Second, the patient group admitted to the intensive or high care units at the time of consultation might be considered MPU-eligible later in their journey through the hospital. An intensive care specialist who participated in this study suggested, some patients may be on a high-care unit due to the current lack of an MPU.

The results of this study could to some extent be generalized to other university medical centers: if they treat complex trauma, carry out complex operations, have a psychiatric ward, and are located in large multicultural cities with community mental care available. However, to determine the need for a local MPU a local needs analysis is recommended. If a hospital has, for example, a dedicated delirium team, the need for an MPU will be very different from that determined this study.

It could be argued that more objective measures are required to determine the need for MPU admission. However, Deson et al. note that professional chart review and close staff contact appear to result in shorter lengths of hospital stay, compared to using formal screening instruments [27]. In future research, the different effects of PCS and MPU admission need to be studied. The value of an MPU should not be confined to a shorter hospital stay or fewer readmissions, because quality of care and reducing the burden on staff caused by disruptive behavior may be equally important arguments for establishing these units. From 2019 onwards, a randomized controlled trial is planned to study the value of the proposed MPU, compared to treatment as usual (PCS only).

## Conclusions

This study shows that a group of patients who currently receive psychiatric consultation (PCS) appears to need admission to a high acuity MPU. This result, based on the judgment of professional medical personnel, supports the initiative of establishing an MPU at the Erasmus MC that must improve diagnosis, (psychiatric) management and safety for medical in-patients with prominent psychiatric symptoms as well as safety risks. The MPU must be equipped to accommodate acute admissions and should be able to cater for patients with complex medical and psychiatric problems in order to provide added value and be acceptable for medical specialists in a tertiary care setting.

## Abbreviations

CI: Confidence interval
FTE: Full-time equivalent
HIV: Human immunodeficiency virus
ICU: Intensive care unit
LCS: Liaison-Consultative Service
LOS: Length of stay
MPU: Medical Psychiatry Unit
OR: Odds ratio
PCS: Psychiatric Consultative Services
